# Apohemoglobin-haptoglobin complex alleviates iron toxicity in mice with β-thalassemia via scavenging of cell-free hemoglobin and heme

**DOI:** 10.1016/j.biopha.2022.113911

**Published:** 2022-10-26

**Authors:** Carlos J. Munoz, Ivan S. Pires, Vinay Jani, Srila Gopal, Andre F. Palmer, Pedro Cabrales

**Affiliations:** a Department of Bioengineering, University of California San Diego, La Jolla, CA, USA; b William G. Lowrie Department of Chemical and Biomolecular Engineering, The Ohio State University, Columbus, OH, USA; c Department of Medicine, University of California San Diego, La Jolla, CA, USA

**Keywords:** Apohemoglobin, Haptoglobin, Iron toxicity, Thalassemia, Anemia, Hemolysis

## Abstract

β-thalassemia is a genetic hemoglobin (Hb) disorder that affects millions of people world-wide. It is characterized by ineffective erythropoiesis and anemia. The resultant chronic anemia can require life-long blood transfusion regimens, leading to secondary hemochromatosis. Moreover, the abnormal red blood cells (RBCs) from β-thalassemia patients are prone to hemolytic events that release cell-free Hb and heme causing a series of events that result in oxidative organ and tissue damage. In this study, β-thalassemic mice were treated with a protein scavenger for six weeks, apohemoglobin-haptoglobin (apoHb-Hp), this protein scavenges cell free Hb and heme. We hypothesize that scavenging cell-free Hb and heme will lead to a positive therapeutic event. After the apoHb-hp treatment it was observed to reduce the weight of the liver and spleen and show an improvement in liver function by a drop in ALT, AST, and ALP markers. ApoHb-hp treatment also hints at an improved RBC half-life as the number of reticulocytes decreased, the mean corpuscular volume (MCV) increased, mean corpuscular hemoglobin increase and the RBC distribution width decreased. Furthermore, apoHb-Hp treatment reduced circulating serum iron concentration and transferrin saturation concentration. Based on these outcomes, introducing a scavenger protein can benefit β-thalassemic mice. This study demonstrated that apoHb-Hp treatment may be a viable strategy to mitigate toxicities associated with cell free Hb and heme, a driver of β-thalassemic issues.

## 1. Introduction

Every year, more than 300,000 children are born with a genetic disorder involving hemoglobin (Hb) [1]. Of these births, approximately 70,000 suffer from thalassemia with more than half of these individuals experiencing severe forms of the disease (β-thalassemia major and HbE β-thalassemia) [1]. In thalassemia, deletions or mutation in genes coding for the α- or β-globin chain of Hb result in a decrease or absent production of one of the globin chains [2,3]. Unfortunately, excess α- or β-chains are unstable and cannot properly transport oxygen, reducing oxygen carrying capacity. Furthermore, the abnormal globin chains can lead to intracorpuscular hemolysis, and, in β-thalassemia, excess α-chains quickly precipitate within the RBC precursors (reticulocytes) in the bone marrow, damaging the erythropoietic system.

Considering the complications originating from defective globin chain production in β-thalassemia, chronic anemia is the most common clinical condition due to the defective production and accelerated destruction of RBCs. Hence, β-thalassemic patients often require frequent blood transfusions [4,5]. Moreover, ineffective erythropoiesis, anemia and accelerated hemolysis induce marked erythroid hyperplasia in the bone marrow, as well as extramedullary hematopoiesis and hepatosplenomegaly [3]. Notably, the constant drive for hematopoiesis and blood transfusion can lead to secondary hemochromatosis (i.e., iron overload) which is the primary cause for morbidity and mortality in thalassemic patients [6]. Since humans have no mechanism to eliminate excess iron, the quantity of iron in the body is carefully regulated primarily via control of gastrointestinal iron absorption. In thalassemic patients, chronic hemolytic anemia increases iron absorption leading to iron accumulation in the spleen, liver, endocrine glands, and heart [7]. When left untreated, iron build-up in the heart can progress towards cardiomyopathy. Furthermore, iron build-up in the liver causes portal fibrosis, cirrhosis, and portal hypertension, which can exacerbate splenomegaly. Unfortunately, the frequent blood transfusion regimens in thalassemic patients further increase iron levels, since one unit of packed RBCs contains approximately 200 mg of iron, while the normal intestinal iron absorption rate is 1–2 mg per day [6,8]. Thus, thalassemic patients receiving blood transfusions frequently require iron chelation therapies to neutralize and eliminate excess free iron [4,5].

In addition, β-thalassemic patients suffer from the toxicities associated with hemolysis. Hemolytic states are characterized by increased levels of circulating cell-free Hb, free heme and iron which are toxic oxidative species [9]. In thalassemia, these toxicities are exacerbated by the frequent RBC transfusions patients receive, which can be up to 25 % hemolyzed after 24 h in circulation, and secondary hyperkalemia and hypocalcemia from the citrate contained in the transfused blood units [10–13]. Under normal conditions, the damaging effects of these toxic molecules are mitigated by naturally occurring plasma proteins such as haptoglobin (Hp) and hemopexin (Hpx) which scavenge cell-free Hb and heme, respectively [9]. However, these scavenging proteins easily become saturated or depleted during chronic or acute hemolytic states, enabling the cell-free Hb and heme to elicit their toxic effects.

Currently there are no FDA approved therapeutics for hemolysis treatment. In addition to severe anemia, clinically severe thalassemia (*β*^0^*/β*^0^, *β*^+^*/β*^0^) can result in jaundice and hepatomegaly secondary to indirect bilirubinemia from excessive erythrocyte destruction; with few therapies having demonstrated improvement in indirect bilirubinemia in thalassemia. A logical approach for hemolysis treatment would be to supplement the patient with the depleted Hb and heme scavenger proteins Hp and Hpx [6,14,15]. However, none of these therapies have been clinically translated, likely due to the complex and costly purification techniques required for their isolation. Moreover, no studies have shown the effect of a dual treatment modality (i.e., dually treat the effects of cell-free Hb and heme) in treating chronic hemolytic conditions such as β-thalassemia, and how such a treatment strategy relates to observed clinical conditions such as hepatosplenomegaly and hemochromatosis. We have recently demonstrated that the apohemoglobin-Hp (apoHb-Hp) complex can serve as a scavenger of both cell-free Hb and heme [16]. Apohemoglobin (apoHb) is the apoprotein obtained via the removal of heme groups from Hb [17]. ApoHb has unoccupied hydrophobic heme-binding pockets with high affinity for heme, allowing it to serve as a heme scavenger [18,19]. Moreover, when bound to Hp in the apoHb-Hp complex, apoHb is more stable and maintains its heme-binding activity. In addition to heme-scavenging, the apoHb-Hp complex can also scavenge cell-free Hb as it can exchange apoHb αβ dimers for holo-Hb αβ dimers. Thus, this single molecular complex is engineered to function as both a Hpx and Hp mimetic, scavenging cell-free Hb and heme during hemolytic states such as β-thalassemia. Notably, we have developed methods for large-scale manufacturing of these proteins enabling potential clinical translation of this novel therapeutic [17,20].

In this study, β-thalassemic mice were treated with 50 μL of apohemoglobin-haptoglobin (apoHb-Hp, n = 8) complex at a dose of 80 mg/kg or PBS (vehicle, n = 8) over a six-week period to investigate its efficacy to alleviate cell free Hb and heme toxicities.

## 2. Methods

### 2.1. Apohemoglobin preparation

The apoHb used in this study was prepared via tangential flow filtration based on the acidic-ethanol heme-extraction procedure as previously described in the literature [17]. The heme-binding capacity of apoHb preparations was approximately 80 %, with less than 1 % residual heme present. This procedure is detailed in a previous publication that leverages HPLC techniques [16].

### 2.2. Haptoglobin preparation

Human Hp was purified from human Cohn fraction IV (FIV) purchased from Seraplex (Pasadena, CA) via tangential flow filtration as previously described in the literature [20]. The final protein solution was composed of a mixture of Hp2-1 and Hp2-2 Hp polymers, with an average molecular weight of 400–500 kDa and > 95 % purity.

### 2.3. Apohb-Hp preparation

ApoHb and Hp are mixed in a batch prior to the experimental protocol. The mixture is created by calculating a volume ratio, mL of Hp per mL of ApoHb. This volume ratio gives the amount of apoHb needed to mix into to each unit volume of Hp (i.e. if calculated value is equal to 2, for each 1 mL of Hp, need to mix with 2 mL of apoHb solution). This ratio was calculated by

$$\text{Volume}\;\text{Ratio}\left(\text{Hp}:\text{ApoHb}\right)=\frac{\frac{\text{Hemoglobin}\;\text{Binding}\;\text{Capacity}\;\text{of}\;\text{Hp}\left(\frac{\text{mg}}{\text{mL}}\right)}{\text{Total}\;\text{ApoHb}\;\text{Protein}\;\text{Concentration}\left(\frac{\text{mg}}{\text{mL}}\right)}}{\left(\frac{\text{Hb}\;\text{Monomer}(\text{Da})}{\text{ApoHb}\;\text{Monomer}(\text{Da})}\right)}$$

The value of the Hb Monomer used is, 16,125 Da and the value of the apoHb monomer is 15,509 Da. The hemoglobin binding capacity and the total apohb protein concentration is used using the methods described elsewhere [16].

### 2.4. Animal model and treatment

Thalassemic mice consisted of C57BL/6 heterozygous for the Hbb β-globin gene deletion (Hbb^td3th/^BrjK) (beta-thalassemia, Jackson Laboratory). Animals were treated every other day for six weeks with the apoHb-Hp complex (Hp 22.5 mg/mL, apoHb 7.5 mg/mL, 50 μL, n = 8), or vehicle (PBS, equal volume as study group, n = 8) via tail vein injection at a dose of 80 mg/kg. Dose were selected based on previous studies [20]. Animal body weight was monitored at alternate treatment days. Animals were sacrificed after the final dose for analysis.

### 2.5. Hematological parameters

Blood samples were obtained at baseline and every two weeks by retro-orbital puncture under isoflurane (2 % for maintenance, Dräger-werk AG, Lübeck, Germany). Complete blood counts (CBC) were measured on an Hemavet blood analyzer (Drew Scientific, Oxford, CT) and confirmed via flow cytometry on selected samples.

### 2.6. Serum iron content and Tf saturation

Serum iron and unsaturated iron-binding capacity (UIBC) were measured in non-hemolyzed mouse serum using an iron and total iron-binding capacity (TIBC) assay according to the manufacturer’s instructions (LabCorp, Burlington, NC). TIBC and transferrin saturation were calculated from the measured serum iron and UIBC.

### 2.7. Determination of tissue iron content

After the experimental end point was reached, half of the mice (n = 4/group) were euthanized and transcardially perfused via the aorta with PBS, and tissue non-heme iron content was determined with a colorimetric method using BPS (4,7-diphenyl-1,10-phenantroline disulfonic acid) as the chromogen. Briefly, 0.2 g of tissues were incubated overnight in a mixture of trichloroacetic (10 %) and hydrochloric (4 N) acids, and 100 μL of supernatant reduced with thioglycolic acid (Sigma-Aldrich) and acetic acid-acetate buffer (pH 4.5). The ferrous iron content was determined spectrophotometrically (535 nm) with the addition of BPS and after 1 hr incubation at 37 °C. The results were expressed as μg iron/g dry tissue weight.

### 2.8. Histology and iron staining

At the end of the study, half of the mice (n = 4/group) were transcardially perfused with PBS followed by perfusion of a fixative solution (4 % paraformaldehyde in PBS). The liver and spleen were harvested and continued to be fixed in the same solution (4 h at 4 °C). Tissues were washed in PBS, and cryoprotected by immersion in sucrose overnight. Tissues were cut and the free-floating sections were stored in cryoprotective solution at 20 °C until processed. Iron was detected using Perls’ staining for non-heme ferric iron (Fe^3+^) followed by 3,3 diaminobenzidine (DAB, Sigma-Aldrich) in methanol. Iron staining was developed by incubation of tissues with DAB and hydrogen peroxide, and then transferred onto gelatin-coated slides, rinsed in PBS, counterstained with hematoxylin, dehydrated and mounted. Quantification of iron positive cells was performed with a high-resolution microscope using a digital CCD ORCA-285 camera (Hamamatsu, Hamamatsu City, Japan). Images for Perls’-stained areas and Hoechst stained areas were prepared using Wasabi Imaging Software (Hamamatsu). The ratio of pixels stained for Perls’ Prussian Blue in each region compared to the total cellular area of the image was calculated. Ten images were analyzed, by sections, and the results were pooled to determine the mean and standard deviation (SD). To indicate colocalization of Perls’ Prussian Blue and Hoechst in cells, positive cells were counted.

### 2.9. Statistical analysis

Results are presented as max and min box plots. Data analysis between groups and time points were analyzed via two-way analysis of variance (ANOVA). Before experiments were initiated, sample sizes were calculated based on α = 0.05, and power = 0.9 to detect differences between primary end points (serum iron and transferrin saturation). All statistics were performed in GraphPad Prism 7 (GraphPad, San Diego, CA). Results were considered statistically significant if p < 0.05.

## 3. Results

This study was completed in sixteen *Hbb*^*th3/*+^ mice. Eight animals were randomly assigned to each experimental group. The first experimental group was untreated, receiving only PBS (vehicle group). The second group was treated with the apoHb-Hp complex (apoHb-Hp group). All animals were confirmed positive for β-thalassemia via genotyping performed by the vendor and tolerated the experiments without signs of pain or discomfort.

**Fig. 1. :**
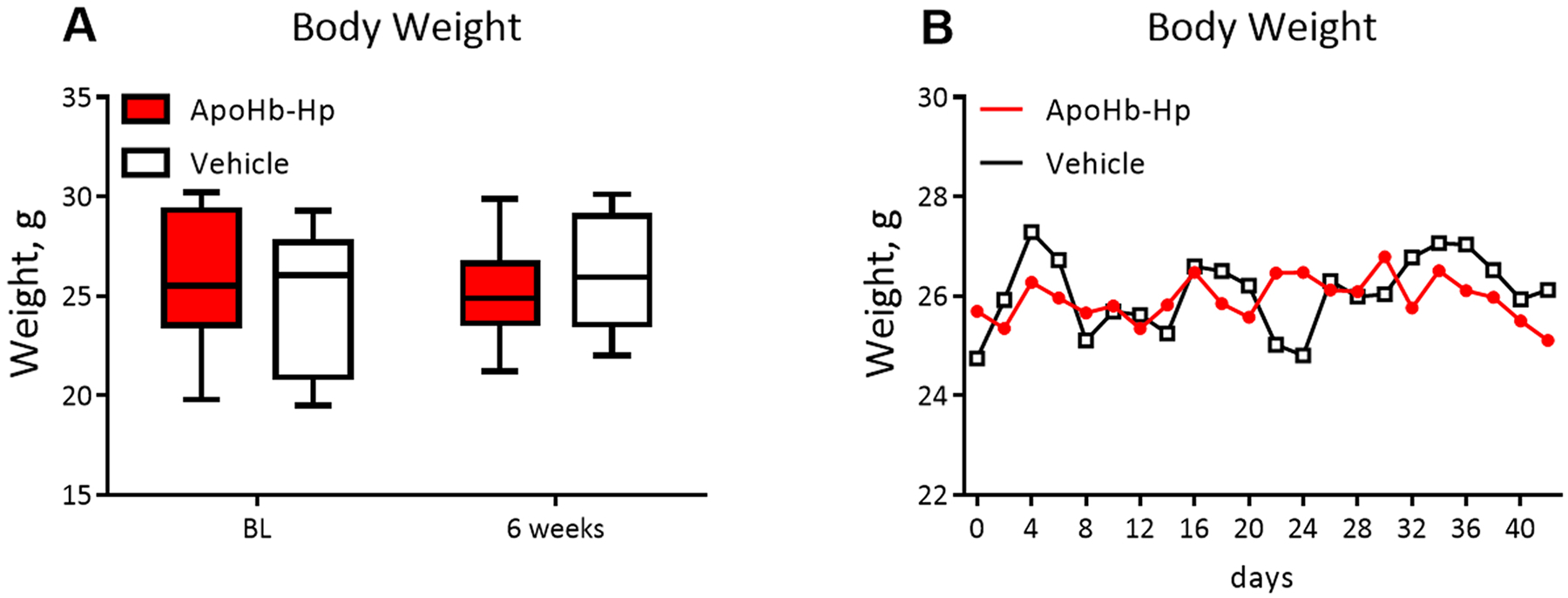
Toxicity of repeated apoHb-Hp administration. (A) Animal body weight at baseline (BL) and after six weeks of treatment with apoHb-Hp or vehicle. (B) Animal body weight tracked continuously over six weeks of treatment with apoHb-Hp or vehicle (Mean is presented; error bars omitted for clarity). N = 8/group.

Six-weeks of apoHb-Hp treatment does not show signs of toxicity. The toxicity associated with continuous apoHb-Hp treatment was assessed by measuring the body weight of animals during the experiment. Fig. 1A shows the body weight of mice at the beginning and end of the study, while Fig. 1B shows the changes in weight of mice every four days during the study. As shown in Fig. 1, the treated and vehicle control mice showed similar weight fluctuations over the length of the study with no significant differences. Moreover, there was no significant change in body weight over the study period for both groups.

**Fig. 2. :**
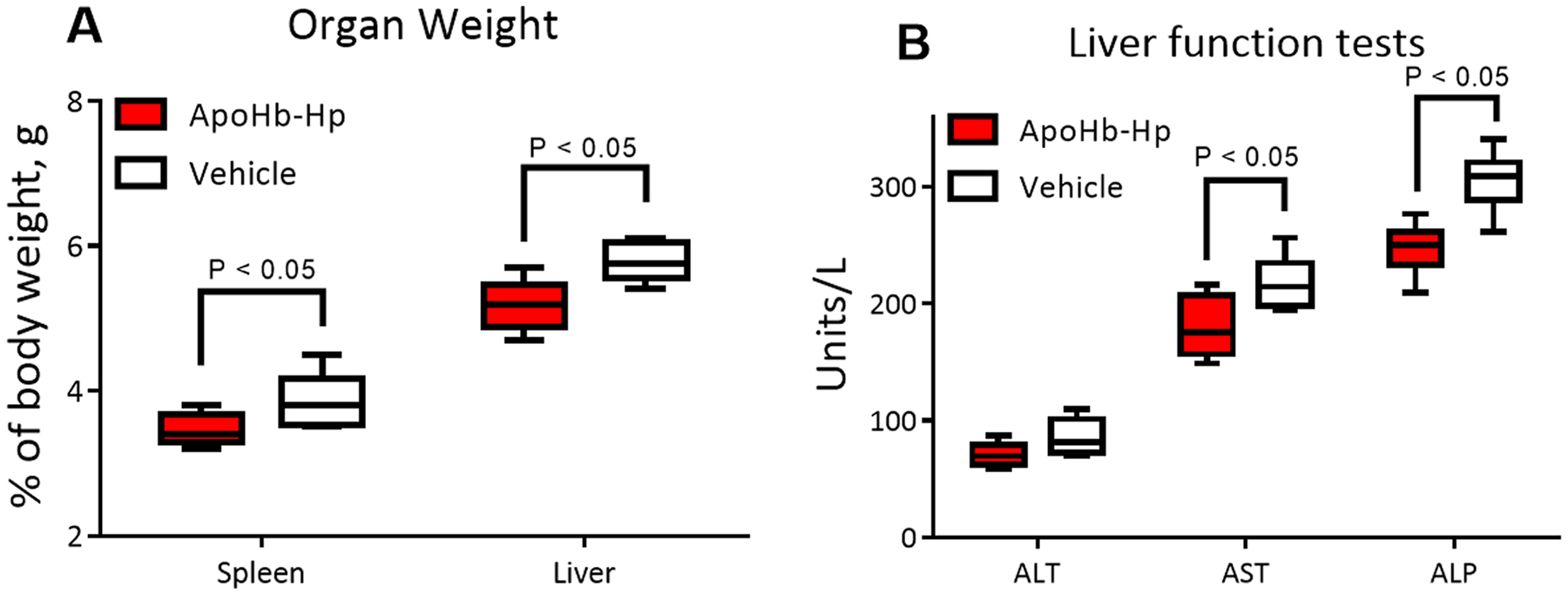
Hepatosplenomegaly and liver function tests. (A) Liver and spleen weight after six weeks of apoHb-Hp treatment compared to the vehicle control. Normal healthy liver and spleen weight for C57BL/6 mice weighing around 25 g according to our laboratory records are 1.2 ± 0.2 g (4.8% of the animal body weight) and 0.7 ± 0.1 g (2.8% of the animal body weight), respectively. (B) Liver function panel focusing on alanine amino transferase (ALT), aspartate amino transferase (AST), and alkaline phosphatase (ALP) after six weeks of apoHb-Hp treatment compared to the vehicle control. Normal healthy ALT, AST, and ALP for C57BL/6 mice according to our laboratory records are 34 ± 8 U/L, 87 ± 14 U/L and 136 ± 22 U/L, respectively. N = 8/group.

ApoHb-Hp treatment reduces splenomegaly, hepatomegaly and markers of liver damage induced by β-thalassemia. The enlargement of the spleen (splenomegaly) and liver (hepatomegaly) are common pathological traits of β-thalassemia [3]. Fig. 2A compares the weight of the spleen and the liver at the end of the experiment for the apoHb-Hp complex treated group and vehicle control group. The spleen and liver weight for the apoHb-Hp complex treated group was significantly lower compared to the vehicle control group. Fig. 2B shows key markers of liver function [alanine amino transferase (ALT), aspartate amino transferase (AST) and alkaline phosphatase (ALP)]. AST and ALP levels were significantly decreased in the apoHb-Hp complex treated group compared to the vehicle control group. ApoHb-Hp treatment also decreased ALT values compared to the control group, but the difference was not significant.

**Fig. 3. :**
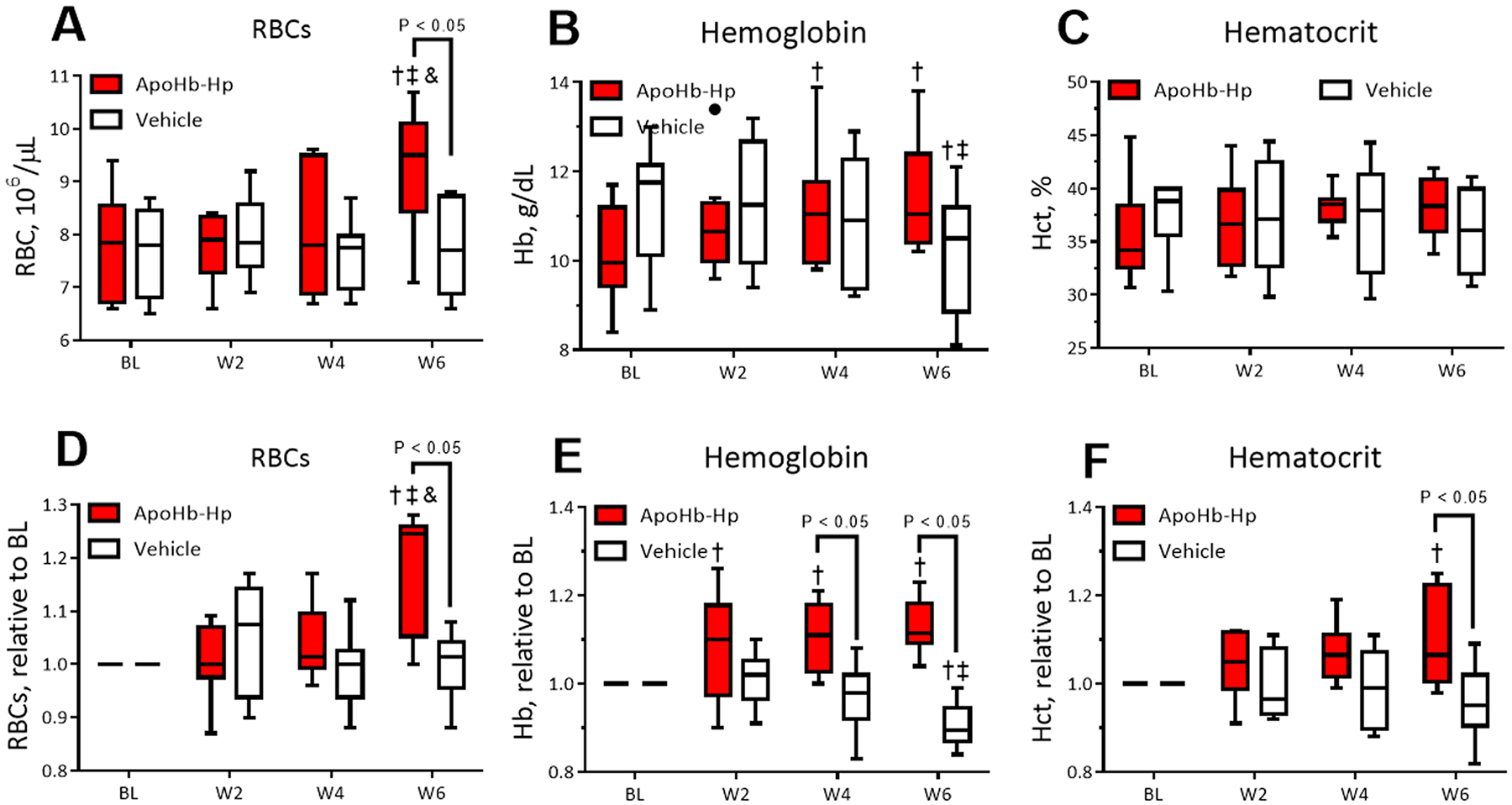
Hematological parameters for RBC and Hb levels. RBC count (A), Hb concentration (B), and hematocrit (C) measured every two weeks starting at baseline (BL) for apoHb-Hp treated animals and the vehicle. RBC count (D), Hb concentration (E), and hematocrit (F) normalized relative to baseline. Symbols: †, compared to BL ‡, compared to week 2 and &, compared to week 4 (P < 0.05). N = 8/group.

ApoHb-Hp treatment recovers RBC levels in β-thalassemic mice. Fig. 3A, B, and C show the RBC count, total Hb concentration (tHb), and hematocrit (Hct), respectively. Fig. 3D, E, and F show these parameters (RBC count, tHb, and Hct) normalized to baseline levels. The absolute RBC count and tHb were increased compared to baseline after six weeks of treatment with apoHb-Hp. Moreover, at the end of treatment, the apoHb-Hp group had higher absolute RBC count compared to the control group, and the control group had decreased tHb levels compared to baseline. Finally, there were no significant differences in the absolute Hct of treated and untreated groups.

When normalized to baseline, the effects of apoHb-Hp treatment were more pronounced as the normalization accounts for the inherent differences between animals. After six weeks of treatment, the relative RBC count, tHb level, and Hct of the apoHb-Hp treated group increased compared to baseline and were significantly higher than the control group. Moreover, the relative tHb level of the apoHb-Hp treated group was significantly higher than baseline and vehicle controls at 4 and 6 weeks of treatment. Finally, the relative tHb levels of the control group decreased over the experimental study, becoming significantly lower than baseline at six weeks. These results indicate that apoHb-Hp treatment improved RBC levels, reducing the severity of anemia in β-thalassemic mice. Although the increase in tHb levels also indicated an improvement over the control, the increase could be an artifact of higher cell-free Hb retention when bound to the apoHb-Hp complex. Moreover, the relative decrease in tHb of the control group is indicative of worsening of anemia in β-thalassemic animals, which was not observed in the apoHb-Hp treated animals.

**Fig. 4. :**
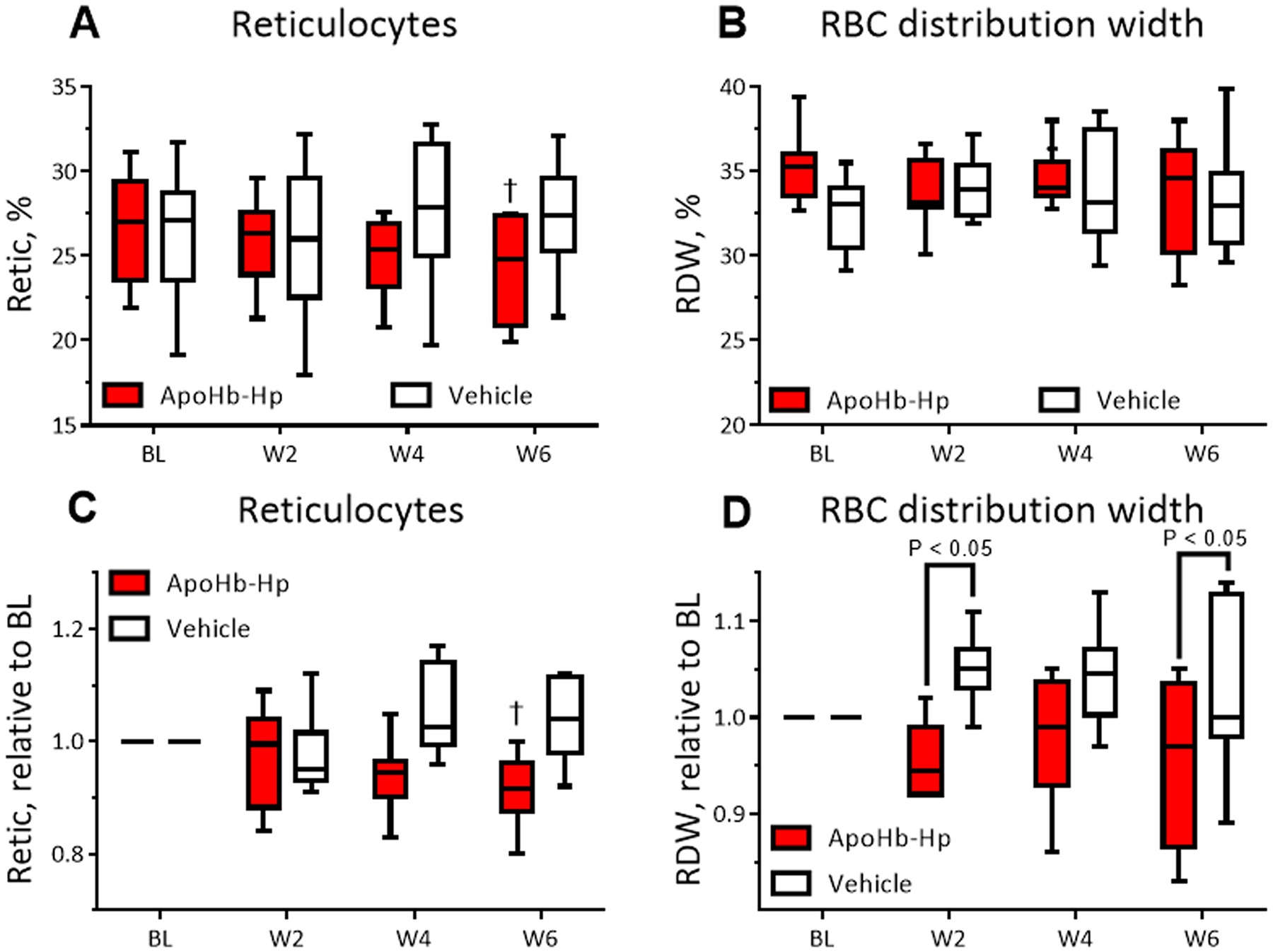
Hematological parameters for RBC turnover rate. Reticulocyte percentage (A) and RBC distribution width percentage (B) measured every two weeks starting at baseline (BL) for apoHb-Hp treated animals and vehicle control treated animals. Reticulocyte percentage (C) and RBC distribution width (D) normalized relative to baseline. †, compared to BL (P < 0.05). N = 8/group.

ApoHb-Hp treatment reduce reticulocytes levels in β-thalassemic mice. The percentage of reticulocytes (Retic) and percentage RBC distribution width (RDW) is shown in Fig. 4A, and B, while Fig. 4C and D shows the Retic and RDW normalized to baseline. Both the absolute and relative change in Retic showed a gradual increase for the control and a gradual decrease for the apoHb-Hp treated group over time. At six weeks, the absolute and relative Retic of the apoHb-Hp treated group was significantly lower than baseline. In hemolytic anemias, the high rate of hemolysis results in higher Retic levels due to the increase in erythropoiesis. Accordingly, apoHb-Hp treatment lowers the drive for erythropoiesis as seen by the increase in tHb and drop in Retic levels. The absolute RDW did not show any significant changes or differences during the six-week treatment. Yet, the relative RDW demonstrated that the untreated group had a significantly higher RDW compared to the apoHb-Hp treated animals at the second and sixth week of treatment. The higher RDW is indicative of the presence of various shaped and deformed RBCs which is consistent with thalassemia [20]. Thus, apoHb-Hp treatment allowed for a normalization of RBC shape in the circulation.

**Fig. 5. :**
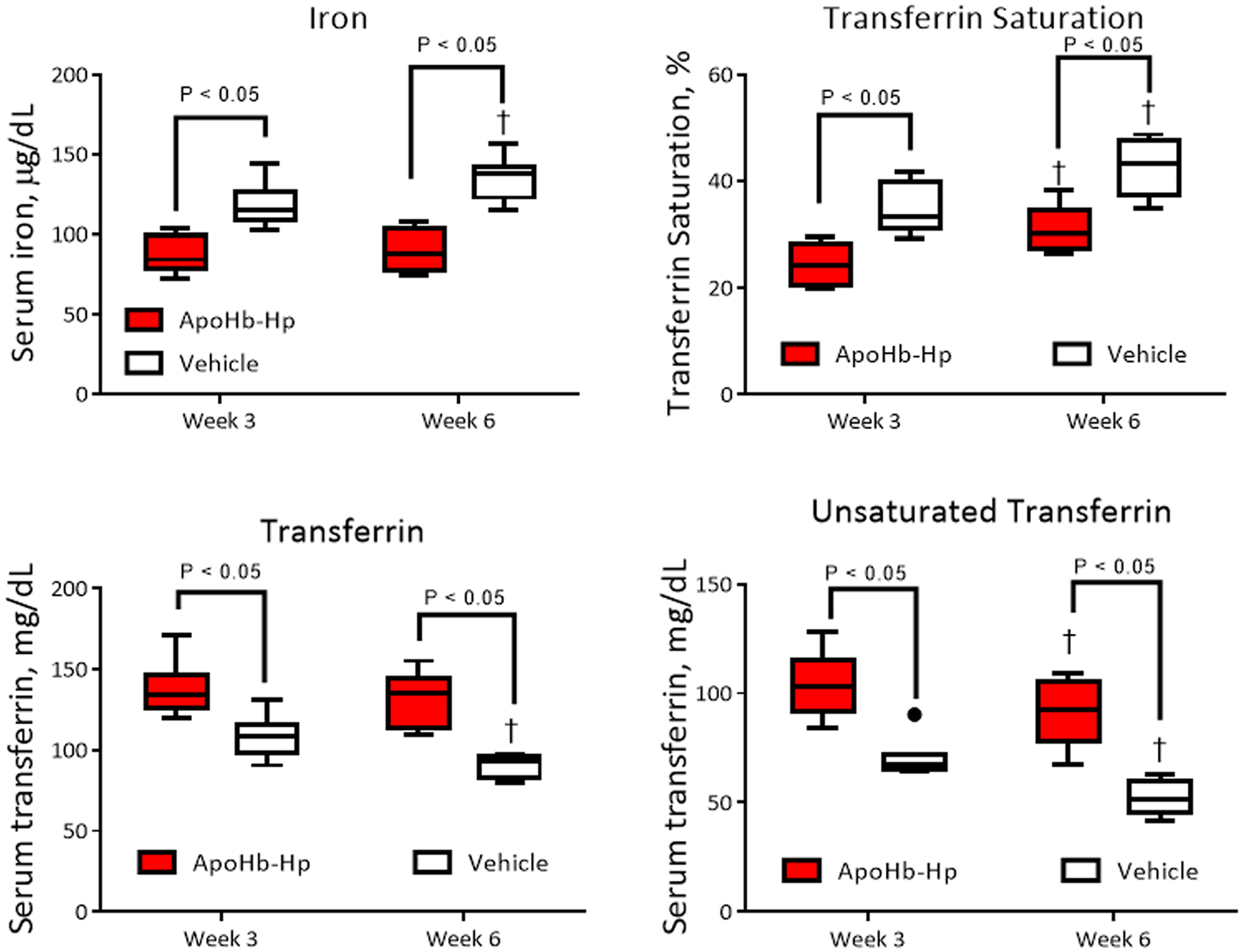
Circulating iron levels. Serum iron concentration (A), transferrin saturation (B), serum transferrin concentration (C) and unsaturated transferrin (D) at the third and sixth week of apoHb-Hp treatment compared to the vehicle control. †, compared to week 3 (P < 0.05). N = 8/group.

ApoHb-Hp treatment lowers total iron levels and increases serum iron binding capacity of transferrin. Fig. 5A shows the serum iron concentration, while Fig. 5B shows the transferrin (Tf) saturation, Fig. 5C shows the serum Tf concentration, and Fig. 5D shows the total unsaturated Tf concentration. The serum iron concentration and Tf saturation in the apoHb-Hp treated group was significantly lower than the control after three weeks and six weeks of treatment. Moreover, Tf levels were significantly higher in the apoHb-Hp treated group. The results also showed that serum iron significantly increased comparing the third and sixth week of the control group, but not for the apoHb-Hp group. Furthermore, Tf levels significantly decreased when comparing the third and sixth week of treatment for the control group. Tf saturation increased for both the apoHb-Hp and control treated groups when comparing the third and sixth week. Lastly, unsaturated Tf significantly increased in the apoHb-Hp treated group. These results showed that apoHb-Hp treatment lowered the free iron load in circulation and increased the total iron binding capacity of the serum, indicating a reduction of hemochromatosis associated with thalassemia.

**Fig. 6. :**
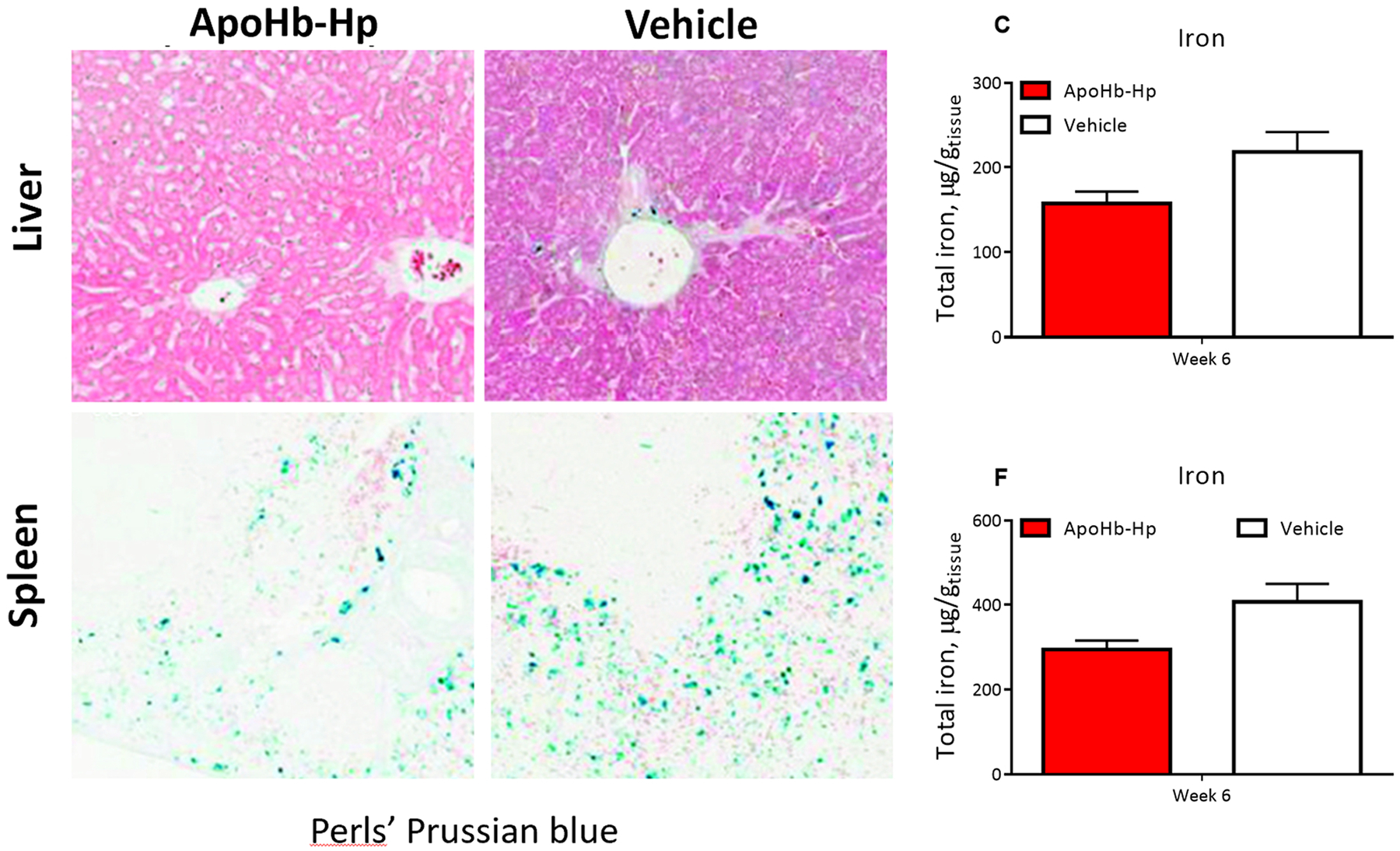
Tissue iron deposition. Representative liver iron staining after six weeks of apoHb-Hp treatment (A) compared to the vehicle control (B). Total iron per gram of tissue in the liver after six weeks of apoHb-Hp treatment compared to the vehicle control (C). Representative spleen iron staining after six weeks of apoHb-Hp treatment (D) compared to the vehicle control (E). Total iron per gram of tissue in the spleen after six weeks of apoHb-Hp treatment compared to the vehicle (F). Blue stained areas represent sites of iron accumulation. Data are presented as mean ± SD. N = 4 animals/group.

ApoHb-Hp treatment reduces iron accumulation in the liver and spleen. Fig. 6 shows iron staining in the liver and spleen after six weeks of treatment. There was a decrease in iron staining in images of both the liver (Fig. 6AB) and spleen (Fig. 6DE) for apoHb-Hp treated animals. When total iron was quantified (Fig. 6C and F), this decrease became more apparent, but the difference was not statistically significant. Therefore, this data corroborates the results shown previously that apoHb-Hp treatment reduces iron accumulation in β-thalassemic mice.

## 4. Discussion

Following preclinical evaluation over a six week regimen of apoHb-hp, it was observed that reducing the amount of cell free hemoglobin and heme in β-thalassemic mice correlates to a drop in spleen and liver weight, drop in AST, ALT and ALP concentrations, a drop in RBC distribution width, a drop reticulocyte count, an increase in both mean corpuscular volume and hemoglobin, a drop in serum iron concentration and a drop in transferrin saturation percentage. The drop in spleen and liver weight, and drop in AST, ALT and ALP concentrations are associated with better organ function. A drop in RBC distribution width, a drop in reticulocyte count and increase in both mean corpuscular volume and hemoglobin are associated with increase in RBC half-life. A drop in serum iron concentration and a drop in transferrin saturation percentage are associated with a better regulation of iron. An interpretation of these results are that increasing the concentration of scavenger proteins decreases iron accumulation in the spleen and liver from excess RBC turnover, leading to better organ functionality in mice with β-thalassemia. One hypothesis to explain the promising results are that apoHb-hp lowers work hypertrophy and extramedullary hematopoiesis known to induce enlargement of these organs. Briefly, work hypertrophy and extramedullary hematopoiesis results from either an increased activity of splenic macrophages in response to thalassemic erythrocytes [21,22] or to chronic anemia and tissue hypoxia, where erythroblast production is increased at sites of fetal erythropoiesis, namely the liver and spleen. The latter of these is believed to be the primary mechanism for hepatomegaly in thalassemia [23–27]., We hypothesize apoHb that scavenging cell-free Hb and heme reduces vascular dysfunction and increasing RBC half-life alleviating chronic anemia and tissue hypoxia. Cell free hb and heme are known to increase intravascular oxidative stress because they trigger a cascade of events: nitric oxide scavenging, a release of inflammatory cytokines, a release of adhesion proteins, and an increase in reactive oxygen species (ROS) [28–31]. Thus, controlling the concentrations of cell free hemoglobin and heme can prevent the negative downstream affects known to cause vascular injury and cell death thus improving anemic states. Considering the former, a decreased activity of splenic macrophages by apohb-hp despite thalassemic erythrocytes, is due in part to the reduction of RBC sequestration in the spleen [32]. Previous studies have demonstrated that vaso-occlusion induced by hemolysis can increase capture of RBCs within the spleen [33]. Thus, by reducing the hemolysis induced side-effects of β-thalassemia, apoHb-Hp treatment may reduce RBC capture, leading to the observed decrease in the size of the organ.

Both hypotheses associated with splenomegaly point to an increase RBC half-life. As previously mentioned by lowering the concentration of free hemoglobin and free heme you may lower the concentration of other proteins, cytokines and enzymes that may cause oxidative damage to the vasculature, but also to the RBCs themselves. Oxidative damage on the RBC membrane induces hemolysis by activating phospholipid scramblase and increasing the exposure of phosphatidylserine (PS) on the membrane of the RBC initiating cell death [34]. Apohb-hp may decrease the concentration of proteins causing oxidative stress. More importantly, regardless of the mechanism the reduction of splenomegaly is vital for clinical management of thalassemic patients. Both extra-medullary hematopoiesis and RBC sequestration strain spleen function which can lead to a hyperactive spleen (hypersplenism). In hypersplenism, the spleen destroys RBCs at an accelerated rate, exacerbating anemia and increasing transfusion requirements. Treatment at severe stages of hypersplenism primarily consists of splenectomy, which can make patients more susceptible to infection and sepsis [35,36].

In addition to splenomegaly, β-thalassemic patients commonly suffer from hepatomegaly (enlarged liver). Similar to splenomegaly, extra-medullary hematopoiesis can also occur in the liver, leading to its enlargment [25]. Furthermore, the increased liver iron pool in β-thalassemic patients is associated with cellular toxicity and its enlargement [23,24]. Notably, the hemolytic environment associated with β-thalassemia can lead to excess heme uptake within the liver, resulting in liver damage and congestion due to inflammation and oxidative stress [26, 27]. This causes chronic liver injury and fibrosis in β-thalassemic patients [37]. One of the consequences from extensive liver injury and fibrosis is portal hypertension, which can contribute to splenomegaly and increases the risk of hypersplenism [38,39]. Moreover, liver disease can also lead to abnormal RBCs as the liver controls lipid metabolism [40]. Thus, the damaged liver and excess hemolytic species (i.e., Hb and heme) can lead to abnormal RBCs, further increasing congestive splenomegaly and anemia. With apoHb-Hp treatment, both extra-medullary hematopoiesis and hemolytic stress can be reduced, leading to normal liver function. As mentioned previously, one of the causes of splenomegaly in β-thalassemic patients is work hypertrophy mediated by thalassemic erythrocytes. In β-thalassemia, these abnormalities in RBCs are not only derived from the genetic deficiency in β-globin production, but hemolytic species (i.e. Hb, heme, and iron) can also lower the lifespan of RBCs. In β-thalassemia, there is an excess of free alpha chains and a release of intra-erythrocytic heme which triggers a hemoglobin oxidation pathway, thus causing damage to the RBCs [41]. With beta-thalassemia, auto-oxidation reflects the formation of superoxide radicals (0_2_) and hydrogen peroxide (H_2_O_2_) resulting in oxidative stress. This occurs due to exposure to high levels of ROS, resulting in oxidative damage to the RBC membrane, leading to premature macrophage capture [42–45]. Therefore, apoHb-Hp treatment may also contribute in reducing the oxidative stress experienced by the liver and RBCs in thalassemic animals, leading to the observed clinical benefits from apoHb-Hp treatment.

**Fig. 7. :**
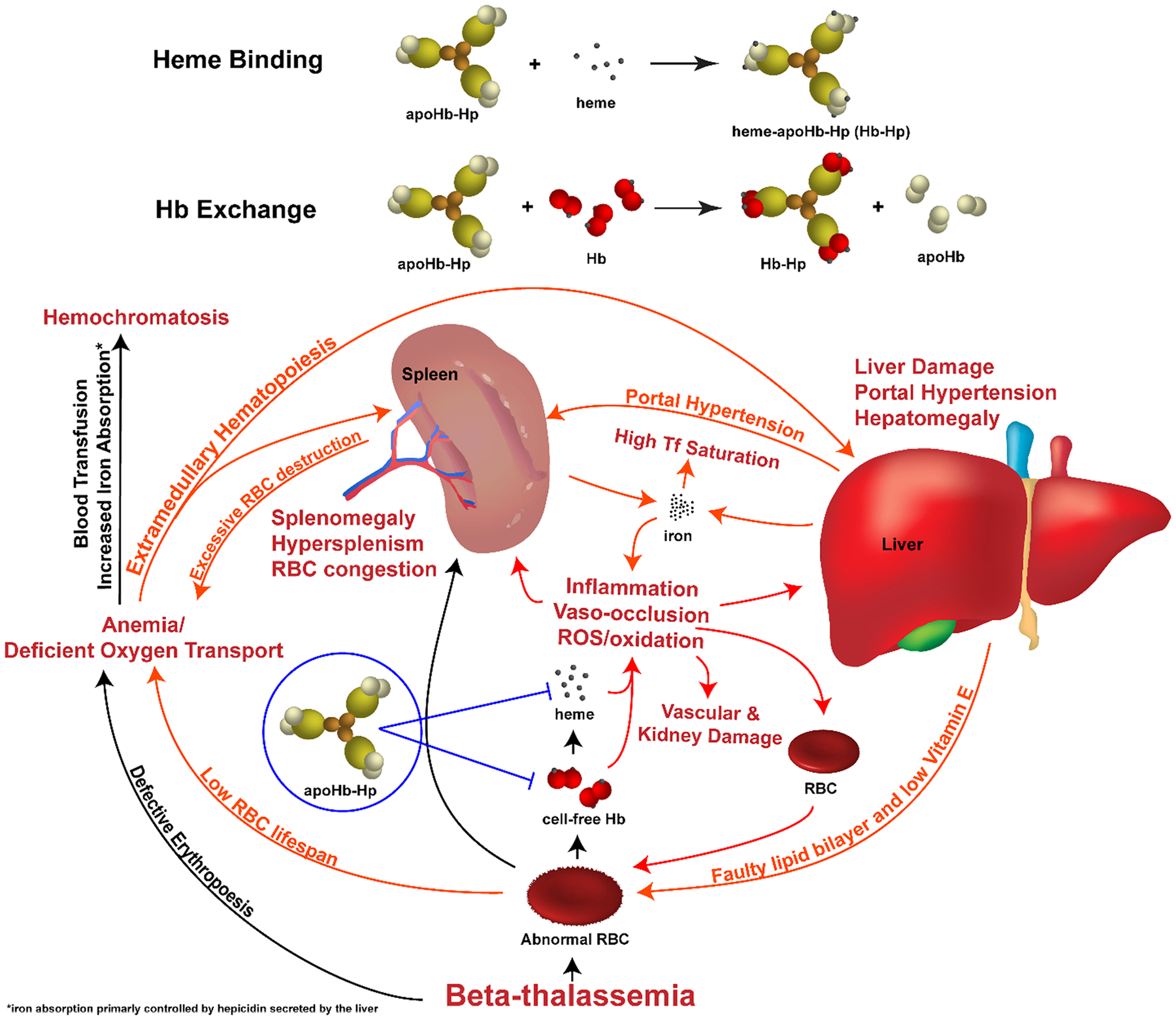
Mechanism of action in apoHb-Hp treatment of β-thalassemia. ApoHb-Hp scavenges cell-free Hb and heme, which prevents the cascade of effects that lead to more severe spleen and liver damage and associated pathophysiology. Red lines indicate the effects of cell-free Hb and heme directly reduced upon apoHb-Hp treatment. Orange lines indicate the secondary effects of apoHb-Hp treatment.

Furthermore, serum iron studies confirmed that treatment with apoHb-Hp resulted in decreased erythrocyte turnover, as evidenced by decreased serum iron, decreased transferrin saturation, and increased transferrin. These data support the hypothesis that treatment with apoHb-Hp independently reduces splenic extravascular hemolysis thereby reducing anemia. In cases of chronic anemia, there is a physiologic increase in iron absorption from decreased hepcidin inhibition of ferroportin activity. Lowering the rate of erythropoiesis in β-thalassemia is important, since a common effect of the severe anemia in β-thalassemic patients is inhibition of the hormonal regulator of iron metabolism, hepcidin [46]. Hepcidin binds to ferroportin and induces its intracellular degradation. This mechanism protects tissues from iron overload as it prevents iron efflux from iron-releasing cells such as macrophages and duodenal enterocytes (responsible for absorption of iron in the intestine). In normal physiological states, high circulatory levels of iron and inflammation stimulate hepcidin expression to prevent iron overload in tissues by restricting iron to macrophages and duodenal enterocytes. However, during thalassemia, the continuous expression of erythropoietin blocks hepcidin expression, leading to high ferroportin levels and subsequent iron overload due to high levels of iron absorption [42,47]. Since apoHb-Hp treatment normalized RBC levels reducing the erythropoietic drive, the treatment likely normalized hepcidin function, leading to regularization of iron levels in the liver and spleen, and the observed reduction in transferrin iron saturation. In the context of thalassemia, this is clinically problematic in the case of blood transfusion, as repeated transfusions of packed RBCs provide an external source of iron which result in iron overload from decreased hepcidin activity [42,47]. Our results consequently suggest that apoHb-Hp may have increased hepcidin activity relative to vehicle treatment, though we did not measure serum hepcidin. Future directions would be to assess hepcidin activity with treatment with apoHb-Hp treatment. Based on the results shown in this study and the described mechanisms of β-thalassemia induced hepatosplenomegaly, a mechanism of action for apoHb-Hp treatment was proposed and is illustrated in Fig. 7.

Consistent with the data presented here and the proposed mechanism of action for apoHb-Hp, previous studies have demonstrated that Hp and Hpx double knock-out mice suffer from splenomegaly and severe liver inflammation and fibrosis when exposed to hemolytic stress [48]. In these prior studies, splenomegaly was primarily caused by the accumulation of RBCs, which was attributed to free heme, leading to vascular alterations that caused adhesion of RBCs to the endothelium [48]. Additionally, Hpx treatment has been shown to reduce liver damage and endothelial function in β-thalassemia and sickle-cell disease [49]. However, Hpx treatment alone did not improve RBC levels in β-thalassemic mice, unlike the apoHb-hp treatment implemented in our study. Moreover, unlike the results shown in our study, one month of Hpx treatment alone increased iron levels in the liver in β-thalassemic mice. While this increase in liver weight would be favorable since it suggests that the excess-heme was directed to cells specialized in the detoxification of heme, the study demonstrated that Hpx treatment alone could not prevent the morbidities of β-thalassemia.

Of all our observations, the observation that treatment with apoHb-Hp improved total RBC count is the most promising for its potential as a therapeutic. These results suggest that apoHb-Hp may offer an alternative to splenectomy, which is known to result in an increased incidence of bacterial sepsis from decreased IgM production [35,36]. Our study showed that only after prolonged treatment, apoHb-Hp increased Hb, reduced reticulocyte count, and RDW. Briefly, anemia results in an artificially increased RDW and reticulocyte count due to the relative decrease in RBC concentration. More importantly, the observed reduction in reticulocyte count indicates a suppression of erythropoiesis suggesting that treatment with apoHb-Hp improved systemic oxygenation and reduced extravascular hemolysis due to increased circulation time [50]. The murine CD163 does not recognize human Hp, so it is unlikely that the apoHb-Hp complex helps with erythropoiesis by recycling heme for Hb synthesis, thus the increase Hb and Hct has to be due to increase circulation time. In summary, our results demonstrate that treatment of thalassemia with apoHb-Hp improves anemia and reduces RBC turnover without the need for external intervention (e.g. transfusion).

## 5. Project limitations

Finally, there were some limitations existing in this study. The data did not show a direct mechanism responsible for increased RBC half-life since the ApoHn-Hp complex only scavenges free heme and free hemoglobin. We expect that the increase in Hb, RBC counts, and hematocrit in the ApoHn-Hp complex might be related to mitigating the heme and hemoglobin toxicity. Furthermore, the project would have benefited from measuring erythropoietin, heme concentration, and inflammatory markers. Lastly, the project would have benefited from adding two additional groups, mice without any disease and mice treated with equal molars of hemopexin. Unfortunately, the commercial availability of hemopexin is very limited in the amount needed for this study.

## 6. Conclusion

In summary, we have presented preclinical evaluation of apoHb-Hp for treatment of beta-thalassemia major in a mouse animal model. Our study demonstrates that treatment with apoHb-Hp over a 6-week period improved anemia and normalized erythrocyte turnover in thalassemia. Furthermore, we observed that absolute RBC count increased despite the lack of change in Hb and Hct, suggesting that apoHb-Hp treatment reversed extravascular microcytic anemia in thalassemia. These results demonstrate that apoHb-Hp has the potential to treat thalassemia without the need of serial transfusions or splenectomy, the current standard of care. Future directions would be to mechanistically evaluate the physiology of apoHb-Hp treatment, particularly with regard to suppression of splenic macrophage mediated extravascular hemolysis and the role of hepcidin. Additionally, future studies should investigate whether apoHb-Hp has the potential to mediate transfusion induced iron overload in this same population. All in all, the ApoHb-Hp has the aspiration of moving into clinical development, the resolve of this project warrants further investigation as it is promising.

## Data Availability

Data will be made available on request.

## Key points

- Apohemoglobin-haptoglobin complex alleviates iron toxicity and regulates RBC turnover in β-thalassemic mice
- Apohemoglobin-haptoglobin complex reduces hepatosplenomegaly in β-thalassemic mice
- Apohemoglobin-haptoglobin complex increase RBC count, total hemoglobin concentration, and hematocrit over 6 weeks in β-thalassemic mice
- Apohemoglobin-haptoglobin complex decresase the concentration of Iron and increase the concentration of avaliable transferrin over 6 weeks in β-thalassemic mice
